# Analysis of spatiotemporal change characteristics of Poyang Lake from 1984 to 2021 based on GEE

**DOI:** 10.1038/s41598-025-25435-0

**Published:** 2025-11-24

**Authors:** Huangao Qiu, Qiuxi Zhang

**Affiliations:** Gandong University, Fuzhou, Jiangxi China

**Keywords:** Long time series, Poyang Lake, Google earth engine, Normalized difference water index, Spatiotemporal change of surface water, Climate sciences, Environmental sciences, Hydrology, Water resources

## Abstract

This study analyzed of spatiotemporal change characteristics of Poyang Lake from 1984 to 2021 by the dataset of Landsat series of satellite imagery and JRC Global Surface Water based on the Google Earth Engine platform. The normalized difference water index combined with the Otsu method was used to extract the water area. The results indicated that from 1984 to 2021, the interannual variation of Poyang Lake’s water area presented the characteristics of “fluctuation decline—fluctuation rise—overall decline—overall increase”. Additionally, the lake areas in Yongxiu, Xinjian, Nanchang, and Poyang were the primary regions contributing to Poyang Lake’s overall area changes. The seasonal variation of Poyang Lake is obvious in a year, the area in summer was larger than that in winter. Compared with 1984, 0.03% of the water area of Poyang Lake in 2021 disappeared permanently, and 8.45% of the water area changed from permanent to seasonal. Lake area changes were jointly driven by climate change and human activities. The average annual temperature increases, agricultural irrigation, reclamation of surrounding lakes and water conservancy engineering caused the reduction in lake area. Increased annual precipitation and the implementation of environmental protection policies were the main factors for the increases in lake area.

## Introduction

Poyang Lake, the largest freshwater lake in China, plays a critical role in the middle and lower Yangtze River basin due to its seasonal hydrological dynamics, discharge capacity, and water exchange characteristics. It has made significant contributions to flood control and water storage, climate regulation, maintaining ecological balance, and maintaining biodiversity, while also affecting the economic development of surrounding areas^1^. After entering the 21st century, under the dual influence of global warming and increased human activities, the storage capacity of Poyang Lake has declined and the dryness has intensified, resulting in a series of problems such as the gradual shrinkage of the lake area, frequent occurrence of floods and droughts, ecosystem degradation, and reduction of biodiversity^2,3^. Since the 18th National Congress of the Communist Party of China, the country has attached great importance to the management and protection of rivers and lakes. The degradation of the water body of Poyang Lake has been effectively alleviated, and the overall situation is a complex situation of slow degradation and gradual recovery. Therefore, it is urgent to conduct long time series research on the spatiotemporal changes of Poyang Lake to reveal and analyze its evolution characteristics, which will help water resources management and social and economic development.

In recent years, scholars have conducted numerous studies on the dynamic monitoring of the Poyang Lake area, changes in hydrological characteristics, evolution of aquatic vegetation, and evolution driving mechanisms, providing important scientific basis for the management and protection of Poyang Lake^4–7^. Liu Hao took Poyang Lake as the research object, extracted water body information from remote sensing images through the water body index method, and explored the changes in its water area and its causes from 1999 to 2019^8^. Wu Changxue used a series of Landsat remote sensing images from 1973 to 2018 to analyze the changing characteristics of the water area of Poyang Lake during the dry season, and revealed the driving factors of its water area evolution^9^. Tian Biqing interpreted the Landsat remote sensing images through the multi-band pixel value comparison method, and combined with the measured runoff and land use data to monitor and analyze the changes in the water area of Poyang Lake during the flood season. It was found that the lake area showed a decreasing trend during the flood season from 1977 to 2017, and the main driving factors of this evolution were the increase in runoff and human activities^10^. Long time series analysis requires the acquisition, storage and processing of a large number of remote sensing images. Due to the low degree of automation of traditional analysis methods, previous studies have problems such as the use of few images, short time span, and time-consuming and labor-intensive research^11^.

With the help of Google’s powerful cloud processing capabilities and its partnership with NASA, GEE users can easily call, process and analyze massive amounts of remote sensing satellite (such as Landsat, Sentinel, etc.) image data and other earth observation data online, providing new solutions for long time series remote sensing big data research and has been widely used^12^. Therefore, based on the GEE cloud platform, this paper uses the Landsat series satellite image data from 1984 to 2021 and the Joint Research Centre (JRC) global surface water dataset to analyze the long time series change characteristics of the Poyang Lake water body, in order to provide a theoretical basis for the protection and management of Poyang Lake and promote the sustainable development of economic and social water use.

## Study area and data

### Overview of the study area

**Fig. 1:**
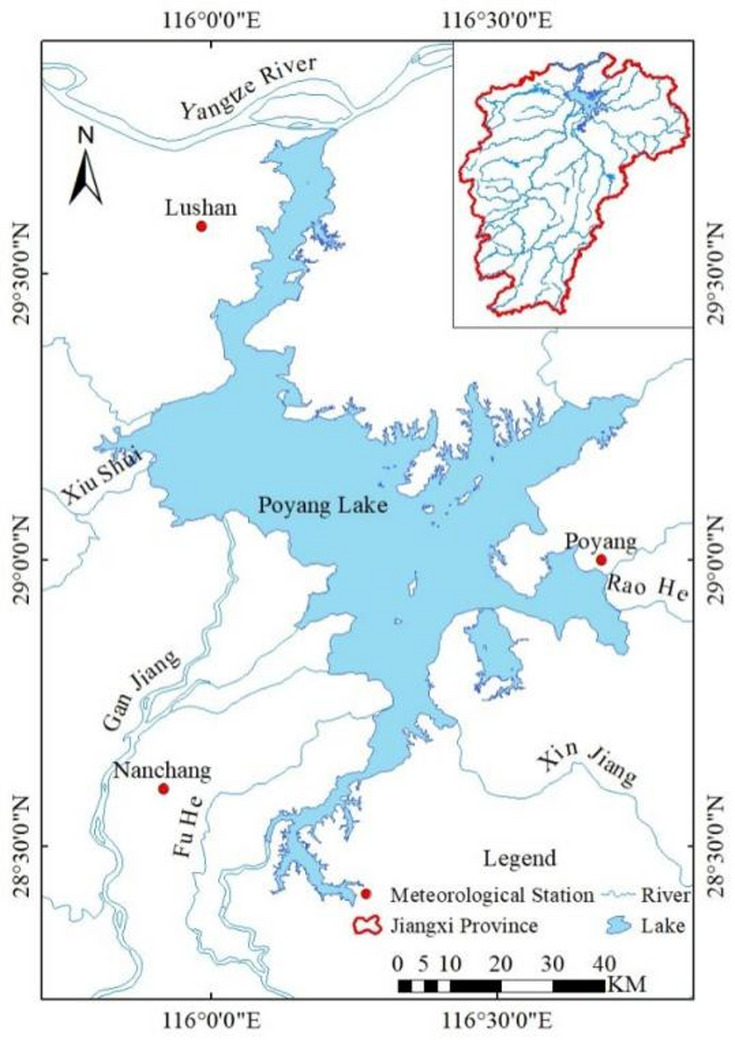
Geographical location of the Poyang Lake. Map created using ArcMap (version 10.6; https://desktop.arcgis.com/en/arcmap/10.6/, Esri, Redlands, CA, USA).

Poyang Lake is located in the northern part of Jiangxi Province (28° 22′ N–29° 45′ N, 115° 47′ E–116° 45′ E). The lake extends 173 km from north to south. It receives water from the five major rivers of Xiu River, Gan River, Fu River, Rao River and Xin River, and injects it into the Yangtze River after regulation and storage. It is a typical lake connected to the river (as shown in Fig. 1, the vector boundary of Poyang Lake comes from the National Science and Technology Infrastructure Platform—National Earth System Science Data Center, the administrative division data comes from the China Geographic Information Public Service Platform). Poyang Lake has a subtropical humid monsoon climate, and the amount of runoff entering the lake has obvious seasonality. Generally, April to September is the flood season, and October to March is the dry season. Since ancient times, it has the characteristics of “floods are everywhere, and low water is one line”^13^.

### Data sources

#### Landsat series satellite imagery

The Landsat series of satellites have continuously observed the Earth for more than 50 years and are the most widely used remote sensing data in long time series research^14^. Therefore, this paper writes code based on the GEE platform to call the Landsat Collection 2 Tier1 dataset as the remote sensing data source. This dataset has undergone systematic radiation correction, geometric correction, and accuracy correction. The following formula (Formula 1, from the United States Geological Survey (USGS) official website) is also required to convert the original digital quantization value (DN value) into the actual surface reflectance value.

1 $$\begin{array}{*{20}{c}} {{\rho _{surf}}={M_\rho } \times DN+{A_\rho }} \end{array}$$

Where ${\rho _{surf}}$ is the surface reflectivity; ${M_\rho }$ is the reflectivity scaling factor; $DN$ is the original digital quantization value; ${A_\rho }$ is the reflectivity offset. For the OLI and TM sensors, follow the surface reflectance band-specific scaling factors provided by the USGS official website. The reflectance scaling factor and reflectance offset are 0.0000275 and − 0.2, respectively. For the MSS sensor, the reflectance scaling factor and reflectance offset of the corresponding band are extracted by reading the metadata file of each image.

**Fig. 2:**
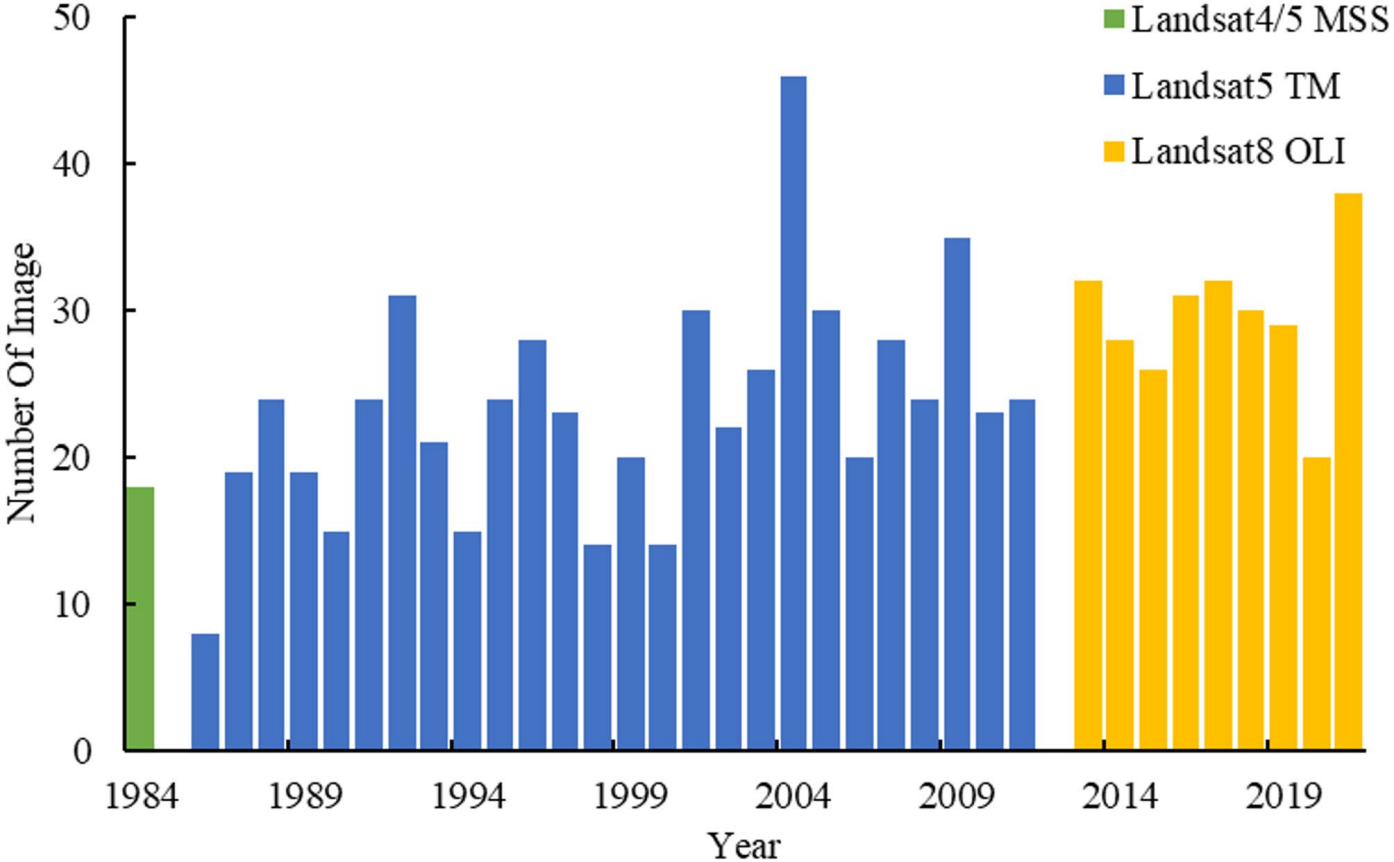
Number of images from different years.

Due to the limited operational lifespan of the satellite, data covering the study area in some years are missing. For example, Landsat4/5 MSS (service life: 1972–1999) lacks data from 1985 onwards; Landsat5 TM (service life: 1984–2012) lacks data from 1984, 1985 and 2012. In addition, images acquired by Landsat7 ETM+ (operating life: 1999–2021) after May 2003 were not taken into consideration because of missing data stripes due to a failure of the satellite’s onboard scan line corrector. Finally, a total of 891 multispectral images of Poyang Lake were ultimately selected for analysis. These images, acquired between 1984 and 2021 from Landsat MSS, TM, and OLI sensors, exclude the years 1985 and 2012 and have a cloud cover of less than 30%. They were used to calculate the water body area. Detailed parameters are presented in Table 1 and Fig. 2.

**Table 1:** Parameters of Landsat series satellite remote sensing images.

Sensor type	Wavelength range (part)/µm	Acquisition year	Spatial resolution/m	Number of image/scene
Landsat4/5 MSS	Band1 (Green): 0.5–0.6 Band4 (Near-infrared): 0.8–1.1	1984	60	18
Landsat5 TM	Band2 (Green): 0.52–0.60 Band4 (Near-infrared): 0.77–0.90	1986–2011	30	607
Landsat8 OLI	Band3 (Green): 0.53–0.59 Band5 (Near-infrared): 0.85–0.88	2013–2021	30	266

#### JRC global surface water dataset

The JRC global surface water dataset in the GEE cloud repository is generated by more than 4.7 million remote sensing images acquired by Landsat5/7/8 satellites between 1984 and 2021. Each pixel of the dataset is classified as water or non-water by the platform using an expert system classifier. The classifier has been verified by more than 40,000 sample points to have a water body misclassification error of less than 1% and a missed classification error of less than 5%. The classification results are organized into monthly data for the entire period (1984–2021) and two periods (1984–1999, 2000–2021) for dynamic change monitoring^15^. This paper uses three bands of the dataset: frequency of water body occurrence, changes in water bodies between two periods, and shifts in water body types during the entire period to analyze the spatial dynamic changes in the Poyang Lake waters.

#### Meteorological data

In order to study the impact of climate on the change of Poyang Lake area, this paper obtained daily observation data from three meteorological stations in different locations around the study area from 1984 to 2021 from the National Meteorological Science Data Center (https://data.cma.cn/), namely Lushan Station, Poyang Station, and Nanchang Station. The geographical distribution of the selected stations is shown in Fig. 1. This dataset contains daily average temperature and daily precipitation. While there is no missing data overall, there are some outliers, specifically manifested as follows: missing values in the data are uniformly filled with “−9999”, and negative values appear in the rainfall data. Such outliers would introduce biases into the subsequent statistical analysis of meteorological data; therefore, we removed the outliers through a year-by-year screening process. After data preprocessing is completed, the temperature data of each station are first averaged station by station and year by year to obtain the annual average temperature of each station; the precipitation data of each station are accumulated station by station and year by year to obtain the annual precipitation of each station. Finally, based on the characteristics of the available meteorological data, spatial interpolation was performed using the Inverse Distance Weighting (IDW) method in ArcGIS software, generating the annual mean temperature and annual precipitation data for the Poyang Lake region from 1984 to 2021.

## Research methods

### Calculation of normalized difference water index

The Normalized Difference Water Index (NDWI) was proposed by McFeeters^16^ in 1996 and has been used ever since. It can effectively suppress vegetation information, weaken the influence of bare soil, buildings, etc., and highlight water information^17^. It is one of the most widely used methods for extracting water bodies based on remote sensing images. Referring to the previous research results, for example, in the study of Ji Mengfei^18^, a variety of water body index methods were selected to compare the water body extraction effects of Landsat remote sensing images of Poyang Lake in different years and seasons. The results showed that the average overall classification accuracy and Kappa coefficient of NDWI were 0.93 and 0.80, respectively, which is the optimal index for water body extraction in Poyang Lake. Therefore, this paper uses NDWI to extract water bodies. The calculation formula is as follows:

2 $$\begin{array}{*{20}{c}} {NDWI=\left( {Green - Nir} \right)/\left( {Green+Nir} \right)} \end{array}$$

Where: Green is the green light band; Nir is the near infrared band. The corresponding band numbers of the above two bands in different sensors of Landsat satellite are shown in Table 1. Code is written in the GEE platform to complete the NDWI calculation.

### Calculation of water area

**Fig. 3:**
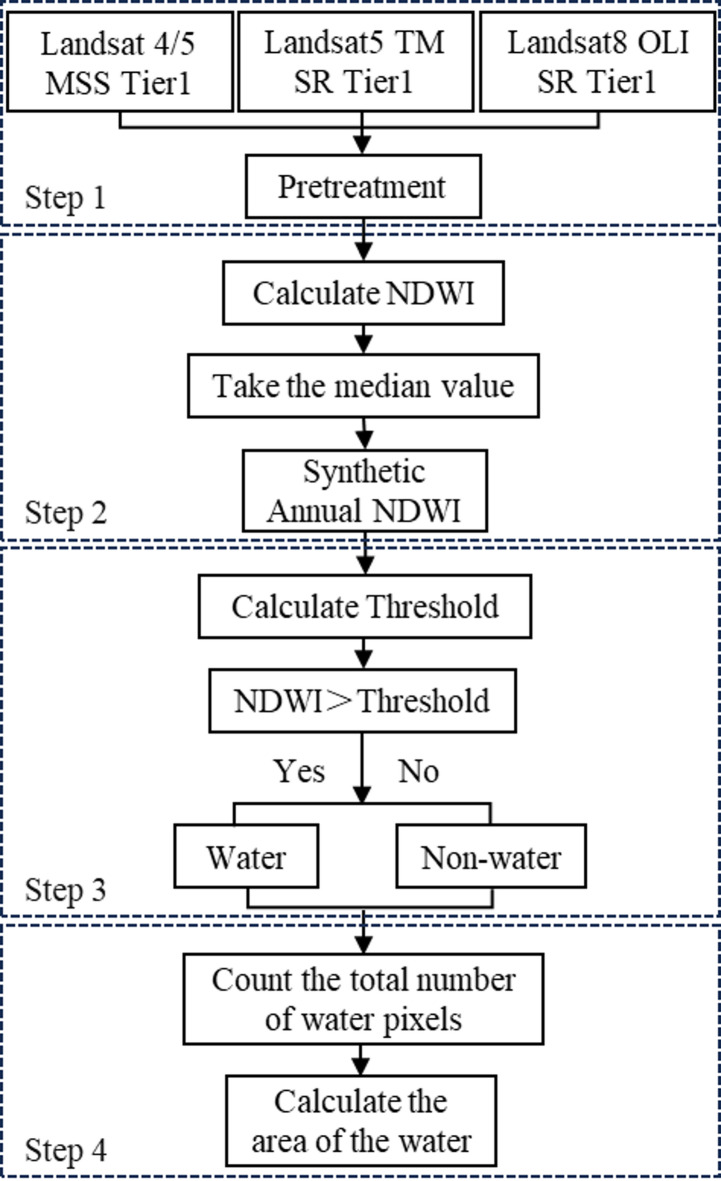
Flow chart of Poyang Lake water area calculation based on GEE platform and Landsat imagery data.

The code was written based on the GEE platform to calculate the lake water area from 1984 to 2021, which includes four main steps. The specific process is shown in Fig. 3.

#### Screening and preprocessing of image data

This paper selects the Landsat4/5 MSS Tier1 and Landsat5 TM/Landsat8 OLI surface reflectance (SR) Tier1 datasets on the GEE platform, filters out the Poyang Lake image data with cloud cover less than 30% in the study year. Using the OLI sensor as a benchmark, the green and near-infrared bands of the MSS/TM sensor are spectrally calibrated using a two-step method: spectral response function (SRF) matching and reflectance ratio fine-tuning. This ensures spectral consistency across different sensors in the same band. Meanwhile, performs cloud removal and other processing operations on the acquired images.

#### Synthetic annual NDWI

Select the corresponding green light and near-infrared bands according to Table 1, calculate the NDWI value for each pixel of the preprocessed image, and then calculate the median value of each pixel year by year to synthesize annual NDWI data.

#### Calculate threshold and extract water bodies

This paper combines the Otsu^19^ method to automatically calculate the segmentation threshold of annual NDWI data. When the NDWI value of a data pixel is greater than the threshold, it is classified as a water body, otherwise the data pixel is classified as a non-water body.

#### Calculate the area of the water body

Count the total number of water pixels within the boundaries of Poyang Lake and calculate the area per unit pixel was based on the spatial resolution of the image, thereby calculate the annual lake water area value.

The calculation method for the water area of Poyang Lake in the four seasons of the year is similar to that of the annual lake water area. It is only necessary to set the time filtering conditions involved in the above process according to the month intervals corresponding to different seasons in the specific year (3 months in total) to obtain the lake water area values in the four seasons of the specific year.

## Results and analysis

### Temporal dynamic changes in the waters of Poyang lake

#### Annual changes in water area

**Fig. 4:**
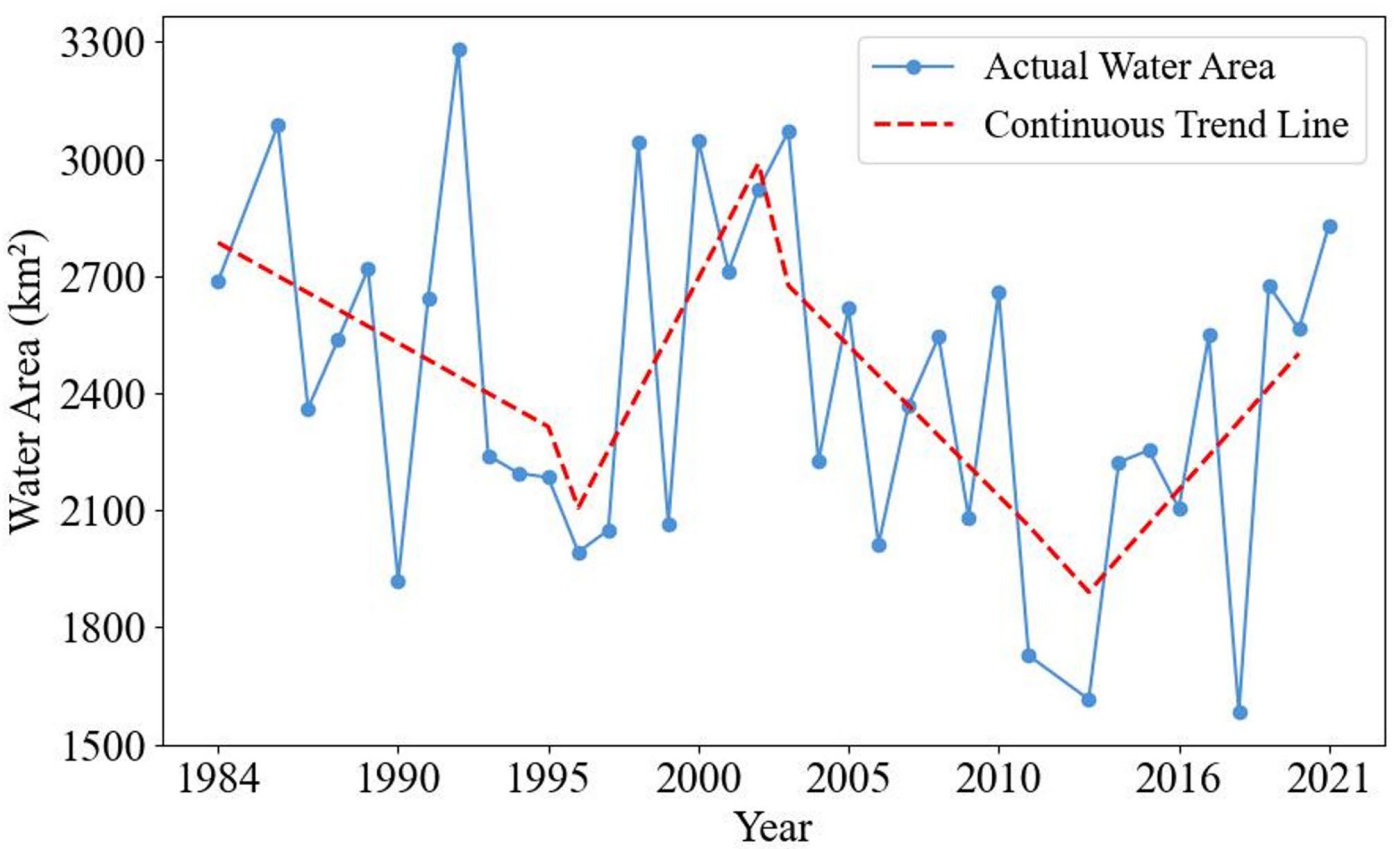
Changes of Poyang Lake area during 1984–2021.

From 1984 to 2021, the interannual changes in the water area of Poyang Lake fluctuated significantly (as shown in Fig. 4). Among them, the maximum water area (3281.75 km²) occurred in 1992, while the minimum (1582.99 km²) was recorded in 2018. Analysis using a five-year sliding mean as the local baseline shows that the water area in 1992 was 33.61% higher than the benchmark for the same period, while the water area in 2018 was 31.04% lower than the benchmark for the same period. Further analysis reveals that the changes in the water area of Poyang Lake are closely associated with the implementation years of national wetland protection policies and large-scale water conservancy infrastructure projects. Therefore, the piecewise linear regression method is adopted to roughly divide the variations in Poyang Lake’s water area during 1984–2021 into four phases. First, the period from 1984 to 1996 was characterized by fluctuation and an overall declining trend. Although the water area in 1986, 1988, 1989, 1991 and 1992 increased to a certain extent or even significantly compared with the previous year, and contained the maximum value in the time series, but the lake area exhibited a decreasing trend, with the water area decreasing by 850.30 km^2^. Following this fluctuation and decline phase, the period from 1996 to 2003 saw fluctuation alongside an overall rising trend, and this stage was generally a period of high water levels for the lake. Specifically, the water area in 1998, 2000 and 2003 all exceeded 3000 km^2^. The average water area during this period was 273.90 km^2^ greater than the overall mean of 2427.65 km^2^. The water area in 2003 increased by 1078.48 km^2^ compared with 1996. Next came an overall decline period from 2003 to 2013, which was generally a period of dry conditions. The average water area during the period was 221.87 km^2^ lower than the overall mean of 2427.65 km^2^, and the overall downward trend was significant. The water area decreased by 1456.22 km^2^, with an average annual decrease of 121.35 km^2^. Finally, the period from 2013 to 2021 marked an overall rising trend. Although this stage included the minimum value in the time series, the overall upward trend is significant, the water area increased by 1215.70 km^2^, and the average annual increase reached 135.08 km^2^.

#### Seasonal changes in water area

**Fig. 5:**
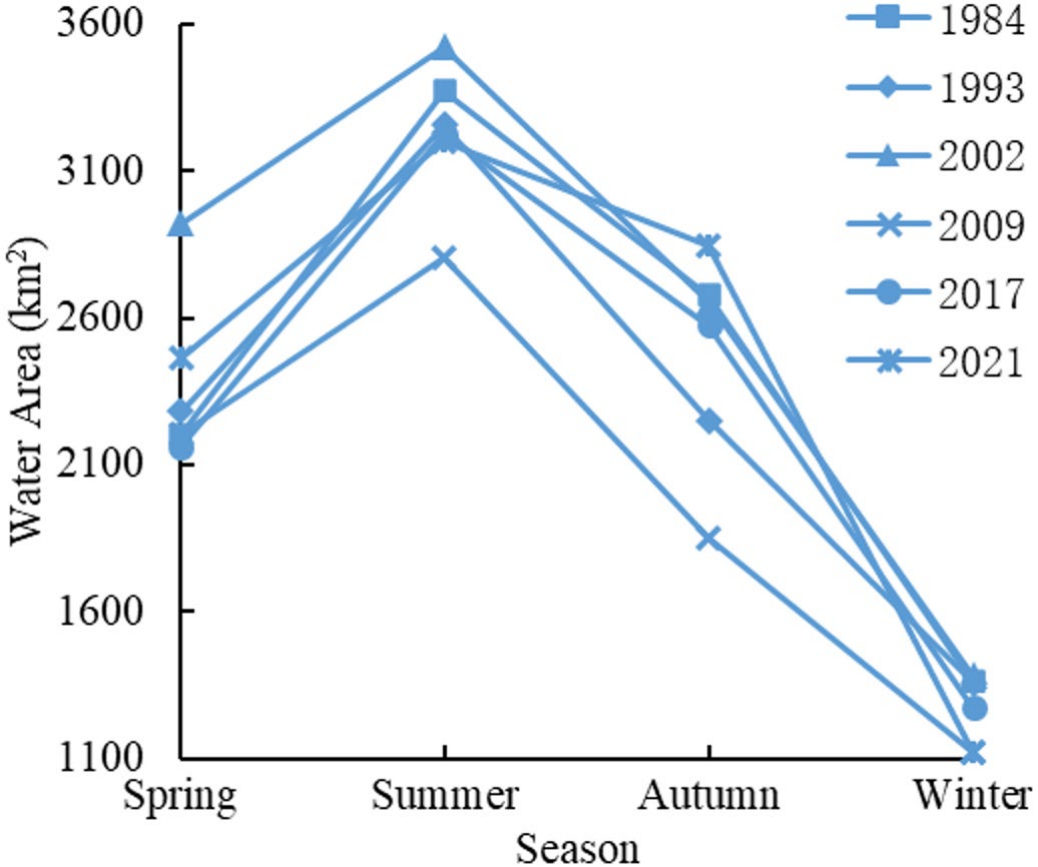
Seasonal change of Poyang Lake area.

In order to deeply analyze the changes in the area of Poyang Lake in four seasons of the year, the water area of Poyang Lake was counted in spring (March to May), summer (June to August), autumn (September to November), and winter (December to February). Due to the missing or poor quality of image data in some seasons in some years, 1984, 1993, 2002, 2009, 2017 and 2021 were finally selected for the seasonal changes in the water area within the year. The results are shown in Fig. 5. Overall, the water area of Poyang Lake shows very obvious changes in abundance and dryness throughout the year: Spring is the rising water period, and the lake water area gradually expands; summer is the high water season, and the lake water area value exceeds 3000 km^2^ at its maximum; autumn is the receding season, and the lake water area gradually decreases; winter is the dry season, and the lake water area value is less than 1500 km^2^ at its minimum.

### Spatial dynamic changes in the Poyang Lake area

**Fig. 6:**
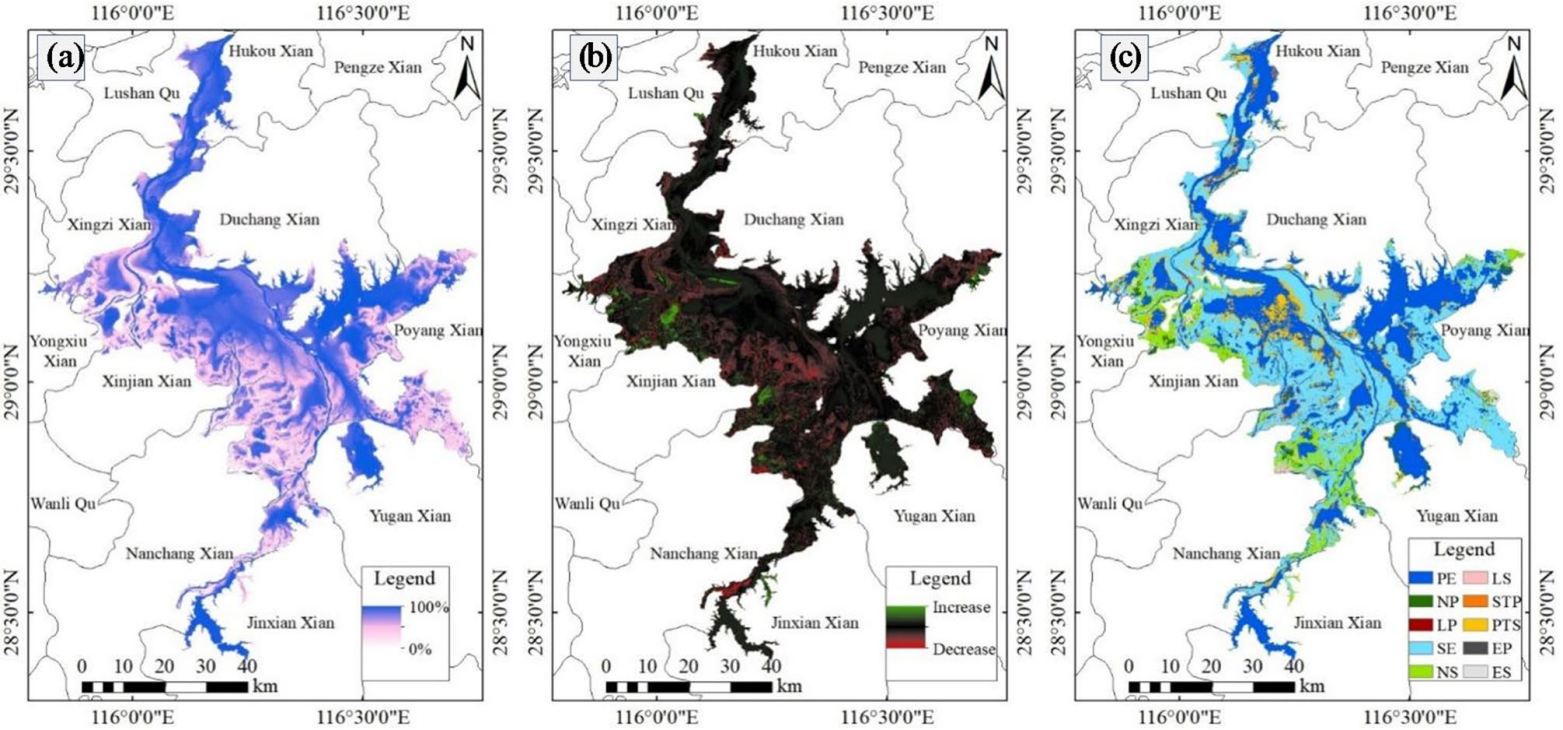
Surface water spatial dynamics in Poyang Lake 1984–2021. **a** Water body occurrence frequency. **b** Changes in water bodies. **c** Water body transfer types. Maps created using ArcMap (version 10.6; https://desktop.arcgis.com/en/arcmap/10.6/, Esri, Redlands, CA, USA).

During the period from 1984 to 2021, the area of permanent water bodies in Poyang Lake was only 34.09%, mainly distributed in Jiujiang City, with small parts scattered in Shangrao City and Nanchang City (as shown in Fig. 6a). As can be seen from Fig. 6b, during the period from 1984 to 2021, changes in the water area of Poyang Lake occurred mainly in Yongxiu County, Xinjian County, Nanchang County and Poyang County, with both a large-scale decrease in area and a relatively obvious increase in area. In terms of water body transfer (as shown in Fig. 6c), during the period 1984–2021, the new and reduced permanent water bodies in Poyang Lake were 1.31% and 0.03% respectively, the new and reduced seasonal water bodies were 7.98% and 0.79% respectively, and 1.43% and 8.45% of the water bodies changed from seasonal to permanent and from permanent to seasonal respectively.

### Factors affecting changes in the water area of Poyang Lake

#### Climate change factors

**Fig. 7:**
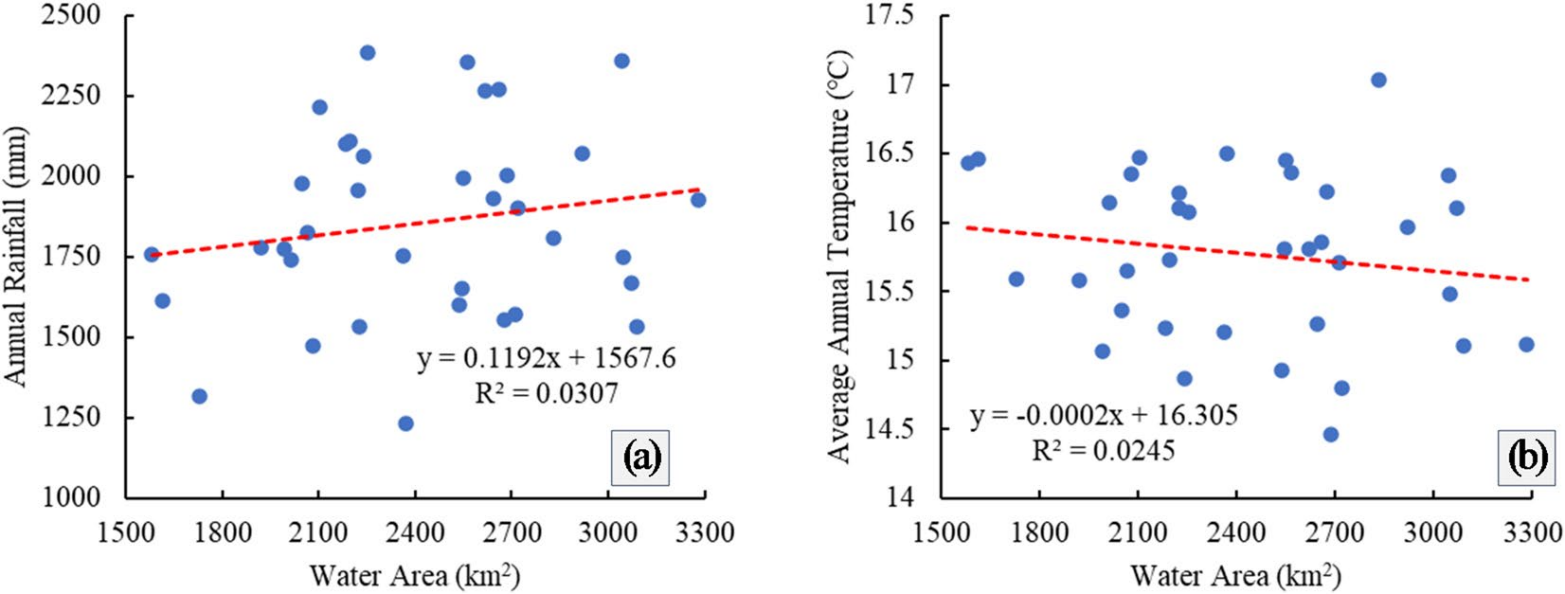
Climate change in Poyang Lake and relationship between in Poyang Lake and climate during 1984–2021. **a** The relationship between water area and precipitation. **b** The relationship between the area of water bodies and temperature.

Under natural factors, climate change directly affects water cycle changes in lake basins. The increase in temperature will promote the evaporation of lake surface water. The increase in precipitation will directly replenish the water volume of lakes, while increasing the runoff of rivers entering the lakes and indirectly replenishing the water volume of lakes, affecting the water area of lakes. The results of Pearson correlation analysis show that during the period 1984–2021, the water area of Poyang Lake was positively correlated with the annual precipitation in the corresponding years, with a correlation coefficient of 0.175; and negatively correlated with the average annual temperature in the corresponding years, with a correlation coefficient of − 0.156, both indicating that the correlation was not significant. Figure 7a and b also verify this analysis.

#### Human activity factors

Under human factors, human activities can change the water cycle process of lake systems. The reason for the fluctuation of the Poyang Lake water area from 1984 to 1996 may be that agricultural irrigation consumes water into the lake, and on the other hand it may be that the phenomenon of enclosing the lake for aquaculture encroaches on the lake water area. Since 1998, the country has gradually implemented the policy of “returning farmland to lakes”. Based on the actual situation, the Poyang Lake basin has responded positively, resulting in an increase in the area of the lake. Since 2003, the Three Gorges Project located in the upper reaches of the Yangtze River has gradually implemented water storage projects, which has weakened the supporting effect of the lower reaches of the Yangtze River on the Poyang Lake area, which may be the main reason for the overall low and dry state of Poyang Lake from 2003 to 2013. Since 2012, the Jiangxi government has begun to implement an ecological migration policy along Poyang Lake, which mainly includes measures such as prohibiting land reclamation around the lake. From 2003 to 2013, the water area of Poyang Lake has generally shown an increasing trend, which may be related to the emphasis and strengthening of environmental protection efforts.

## Conclusion

Based on the analysis of the temporal and spatial variation characteristics of Poyang Lake water area based on the GEE platform, the results show that in terms of temporal dynamic change, the water area of Poyang Lake fluctuated significantly during the period of 1984–2021, showing the characteristics of “fluctuation decline, fluctuation increase, overall decline, and overall increase”. During the year, the seasonal variation of the water area of Poyang Lake was obvious, with summer being the wet season and winter being the dry season, and the water area in summer was more than twice that in winter. In terms of spatial dynamics, during the period of 1984–2021, the area of Poyang Lake in Yongxiu County, Xinjian County, Nanchang County and Poyang County changed significantly, with a large increase and a large decrease. In terms of water transfer, 1.31% and 0.03% of the new and decreased permanent water bodies in Poyang Lake were respectively, and 8.45% of the water bodies changed from permanent to seasonal. The water area of Poyang Lake is caused by climate change and human activities, and the relationship between lake water area and annual average temperature is negative, and the relationship between lake water area and annual precipitation is positive. Agricultural irrigation, lake aquaculture, and water conservancy projects contributed to the reduction in lake area, and the implementation of environmental protection policies is one of the main factors for the increase of lake water area.

This study uses remote sensing technology to analyze the spatiotemporal variations in the water area of Poyang Lake from 1984 to 2021. However, due to limited time and capabilities, some limitations remain. For example, the spatial resolution of Landsat imagery (30 m) limits the identification of small water bodies. Persistent cloud cover during the rainy season in some years of the study area limited the acquisition of sufficient spring and summer imagery, potentially slightly impacting the reliability of core conclusions regarding interannual and seasonal fluctuations in water area. Subsequent research will collaboratively use multi-source remote sensing data (such as high-resolution images, radar images, etc.), utilize the characteristics of different data, improve the accuracy and efficiency of monitoring dynamic changes in water bodies, and establish a comprehensive, multi-time-effective system for monitoring dynamic changes in water bodies.

## Data Availability

All data generated or analyzed during this study are included in this published article.
